# Editing the microbiome the CRISPR way

**DOI:** 10.1098/rstb.2018.0103

**Published:** 2019-03-25

**Authors:** Gayetri Ramachandran, David Bikard

**Affiliations:** Synthetic Biology Group, Department of Microbiology, Institut Pasteur, Paris 75015, France

**Keywords:** CRISPR, microbiome, genome engineering, probiotics, phages, homologous recombination

## Abstract

Our bodies are colonized by a complex ecosystem of bacteria, unicellular eukaryotes and their viruses that together play a major role in our health. Over the past few years tools derived from the prokaryotic immune system known as CRISPR-Cas have empowered researchers to modify and study organisms with unprecedented ease and efficiency. Here we discuss how various types of CRISPR-Cas systems can be used to modify the genome of gut microorganisms and bacteriophages. CRISPR-Cas systems can also be delivered to bacterial population and programmed to specifically eliminate members of the microbiome. Finally, engineered CRISPR-Cas systems can be used to control gene expression and modulate the production of metabolites and proteins. Together these tools provide exciting opportunities to investigate the complex interplay between members of the microbiome and our bodies, and present new avenues for the development of drugs that target the microbiome.

This article is part of a discussion meeting issue ‘The ecology and evolution of prokaryotic CRISPR-Cas adaptive immune systems’.

## 1. Introduction

**Figure 1.**
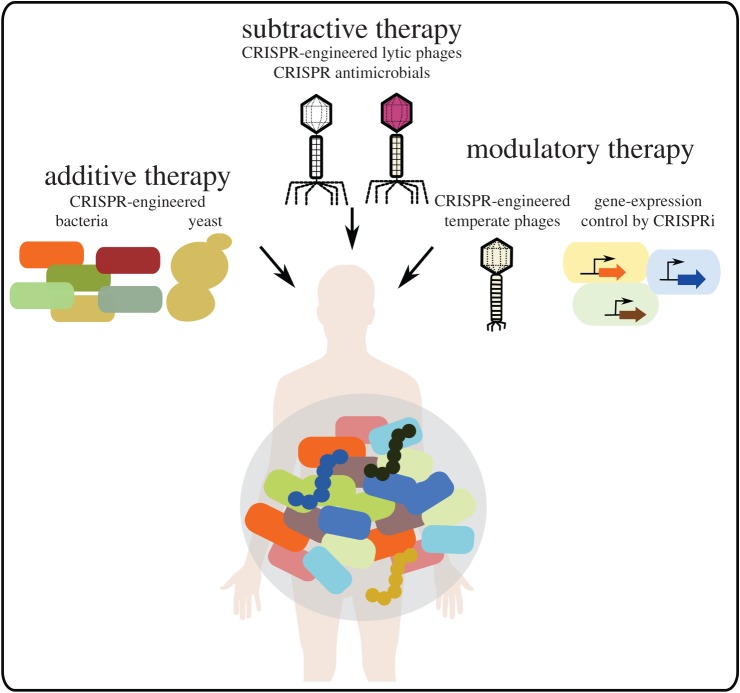
CRISPR approaches to microbiome therapies. CRISPR-Cas systems can be used to engineer designer probiotic strains of bacteria and yeast (additive therapies). CRISPR-Cas systems can also be used to eliminate target bacteria (subtractive therapies), either through the engineering of designer lytic bacteriophages, or through the delivery of CRISPR-Cas systems themselves as antimicrobials. Dead Cas proteins can be used to modify gene expression, and engineered temperate phages can modulate the composition and activity of bacteria in the microbiome (modulatory therapies).

Healthy humans live in a symbiotic relationship with trillions of microorganisms that inhabit the exposed surfaces of our bodies and play an essential role in the maturation of the host-immune response, production of metabolites, brain–gut axis and more (see reviews [1–4]). This close relationship makes our microbiome an interesting target for therapies with the goal to induce desired responses, immunological, metabolic or even neurological in nature. These therapies can be classified into three main types: (i) additive therapies supplementing the host microbiota with individual strains or consortiums of bacterial species, (ii) subtractive therapies aiming to eliminate disease-causing members of the microbiome, and (iii) modulatory therapies aiming to modulate the composition or activity of the endogenous microbiome (see reviews [5,6]). While these therapeutic approaches are still in their infancy, engineered bacteria and viruses can be used to achieve desired outcomes [6]. In this review, we describe how tools derived from the prokaryotic immune system known as clustered regularly interspaced short palindromic repeats (CRISPRs)—and CRISPR-associated (Cas) proteins can be used to modify or eliminate members of the microbiome (figure 1).

CRISPR-Cas systems are the adaptive immune system of bacteria and archaea [7]. The strong interest in these systems comes from the discovery of a set of diverse RNA-guided nucleases able to destroy target nucleic acid sequences, some DNA and other RNA. The Cas nucleases are guided by CRISPR RNAs (crRNA), produced by transcription and processing of the CRISPR locus: a chromosomal site into which DNA fragments from invading nucleic acids are integrated in between repeats, providing a memory of past infections. Cas proteins associated to CRISPR arrays are very diverse and form the basis of the classification of CRISPR-Cas systems into two classes and six main types [8,9]. Class 1 systems (types I, III and IV) consists of a complex machinery, with several Cas proteins assisting the recognition of foreign nucleic acids and their cleavage. Class 2 systems (types II, V and VI) have a simpler protein architecture with a single effector protein arbitrating both recognition and cleavage. The latter class includes the type II CRISPR-Cas9 system, whose versatility has pushed the limits of genome editing [10]. Some features unique to type II systems are the double-stranded (ds) DNA endonuclease Cas9 and the auxiliary trans-acting crRNA (tracrRNA) [11]. The crRNA and tracrRNA can be fused into a chimeric single guide RNA (sgRNA) further simplifying the use of this system as a tool. More recently, Cas nucleases from other subtypes have been successfully used in a variety of biotechnological applications. These include the Cas12 (Cpf1) DNA endonuclease from type V systems as well as the Cas13 nuclease from type VI systems, which targets RNA rather than DNA [12,13].

**Figure 2.**
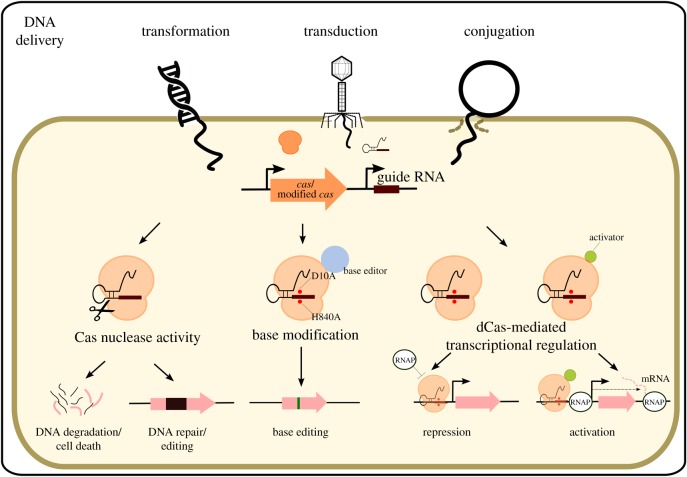
Killing, genome editing or modulation of gene expression by CRISPR-Cas systems. CRISPR-Cas systems can be delivered to target bacteria either *in vitro* or *in vivo* through transformation, transduction or conjugation. Cas nucleases induce DNA breaks that can either lead to DNA degradation and cell death, or if the break is repaired to the introduction of mutations. Catalytically dead Cas proteins such as dCas9 can be used to silence genes by blocking the RNA polymerase (RNAP). Dead Cas proteins can also be fused to various protein domains such as activators to induce the expression of genes, or to domains able to modify DNA bases.

CRISPR-Cas systems are present in approximately 40% of bacteria. Endogenous CRISPR-Cas systems can in some cases be exploited, and engineered CRISPR-Cas systems can otherwise be introduced into target bacteria. These systems can be used to modify the genomes of microbiome-associated or probiotic bacteria, yeast and bacteriophages. They can also be used to kill specific strains based on their sequence without touching the rest of the microbiome (figure 1). Finally, CRISPR-Cas systems can be used to control gene expression without the need to modify the genome. Altogether CRISPR-Cas systems offer a powerful set of tools that will benefit the study of the microbiome and lead to the development of new strategies to modify it (figure 2).

## 2. CRISPR editing of bacteria

Shortly after its discovery and biochemical characterization, the CRISPR-Cas9 system was repurposed to edit eukaryotic and bacterial genomes [14]. It can now be considered as a tool of choice to engineer probiotic strains for additive therapies. Genome editing strategies rely on the use of a guide RNA designed to target a chromosomal sequence of interest where Cas9 will cut. Early bioinformatics studies revealed that bacterial genomes sometimes naturally carry CRISPR-Cas systems that contain guides targeting their own chromosome [15]. In most of these cases clues can be identified showing that the CRISPR-Cas system is inactivated by mutations in the *cas* genes or CRISPR array, or mutations altering the targeted sequences. A model was thus proposed where the CRISPR-Cas systems sometimes capture self-targeting spacers ‘by mistake’ and can only survive such events if the system is functionally inactivated. The idea that self-targeting of the bacterial chromosome by the CRISPR-Cas system is lethal was also corroborated by reports that bacteria die when endogenous or exogenous CRISPR-Cas systems are programmed to target the chromosome [16–19]. While the primary outcome of self-targeting is cell death, some cells are able to survive through the deletion of large DNA fragments encompassing the target position [18,20–23]. This strategy offers little to no control over the extent of DNA that will be deleted, but could still prove useful in removing undesired genetic elements such as pathogenicity islands or prophages from strains of interest. Lethal self-targeting can also be used to counter-select specific genotypes in complex populations [19,24].

The first evidence that CRISPR-Cas9 could be employed to achieve precise scar-less genome editing in bacteria came from a study in which the Cas9 protein from *Streptococcus pyogenes* was integrated in the chromosome of *Streptococcus pneumoniae*, an opportunistic pathogen commonly present in the respiratory tract, sinuses and nasal cavities of healthy carriers [25,26]. In this work, the CRISPR-Cas9 system was programmed to target an antibiotic resistance cassette present in another strain. When the DNA from the first strain was used to transfer the CRISPR-Cas9 system to the second strain through natural transformation, most bacteria died from the activity of the CRISPR-Cas9 system. Nonetheless, a substantial fraction of bacteria survived CRISPR-Cas9 killing through the modification of the targeted position by homologous recombination with the locus present in the donor DNA, which did not carry the antibiotic resistance cassette [26]. This work showed that Cas9 can be used to select for the introduction of mutations at desired positions without the need to leave a selection marker or a scar at the edited position.

While this strategy was easily employed in *S. pneumoniae* where natural transformation and recombination are efficient, its application to less recombinogenic bacteria such as *E. coli* requires the use of the phage lambda red recombination system to promote editing and repair of the Cas9-mediated breaks [26]. Many studies have now expanded on this work, making CRISPR-Cas9 editing tools more convenient to use [27–30]. CRISPR-Cas9 editing strategies typically rely on the expression of guide RNAs, Cas9 and the lambda red genes from one or several plasmids. Template DNA can be provided as short single stranded DNA, short or long double stranded DNA (typically PCR products), or cloned on a plasmid. In all cases Cas9 is guided to introduce a break at a position of interest, which leads to cell death unless the target DNA was modified, or unless it can be repaired by recombination with the template DNA. In a different strategy inspired by previous work with the I-SceI nuclease [31], a non-replicative vector can be integrated through homologous recombination into the locus of interest, followed by Cas9 cleavage of the vector backbone leading to recombination and recovery of the desired scar-less mutation [32,33]. In all the strategies above, steps of plasmid curing can also be necessary and are typically achieved by using the temperature sensitive pSC101 origin of replication [34]. When more than one plasmid is required this can be coupled with other strategies such as targeting the second plasmid with a guide RNA or the use of counter-selection markers such as sacB [27–29].

The fairly large number of components involved, and the necessity to clone a guide RNA as well as in some cases a template DNA, can make these strategies more cumbersome than established methods [35]. CRISPR-Cas9 strategies have nonetheless enabled pushing at the limits of what is possible, in particular where scar-less mutations are needed [27]. Of particular interest, a strategy has been devised to perform high-throughput modifications of many positions in parallel [36]. Pools of oligonucleotides designed to carry both a homologous repair cassette and a sgRNA can be cloned on a vector, yielding a library that can be used to perform multiplexed recombineering. Another clever strategy enabled the replacement of large fragments of the *E. coli* genome with synthetic DNA [37]. In this study, multiple guide RNAs were used simultaneously in the same *E. coli* cell to cleave two positions in the *E. coli* chromosome and two positions on a plasmid carrying a synthetic DNA fragment, triggering the replacement of the chromosomal DNA through homologous recombination.

A novel and powerful approach in the field of genome editing is the use of the catalytic dead variant of Cas9 (dCas9) fused to a cytosine deaminase or an adenosine deaminases in order to convert C•G to T•A or A•T to G•C at specific target positions without the need to introduce a DNA break [38–40]. In this strategy, cytosines or adenosines located within a small window 15–25 bp from the protospacer adjacent motif (PAM) are modified, leading to a somewhat random mutational outcome when several C or A are present in the target window. Nonetheless this approach appears to be very efficient and can easily be used to modify many positions in parallel. In a recent study, Kondo and colleagues optimized this strategy for *E. coli* and were able to modify up to 41 loci simultaneously [41].

CRISPR-Cas9 genome editing tools are already being employed to investigate basic biological questions, as well as in applications such as metabolic engineering [42]. While these applications are not directly related to microbiome engineering, we should keep in mind that *E. coli* is a gut bacterium that can be used as a probiotic. Probiotic *E. coli* strains, like Nissle 1917, have been engineered to express antigens [43], antimicrobial compounds [44], enzymes to disperse biofilms, quorum sensing molecules that control pathogen virulence [45], metabolic functions of interest and more [6]. These current efforts to engineer probiotic *E. coli* strains will certainly benefit from this boon of new tools.

Beyond *E. coli*, the most commonly used probiotic bacteria are Bifidobacteria and Lactobacilli. Engineered Lactobacilli are being developed by various biotech companies as targeted therapies against a wide range of diseases including oral mucositis, inflammatory bowel disease, viral and bacterial infections [46]. The ability of Bifidobacterium to proliferate in solid tumours offers the possibility to engineer them to produce cancer-suppressing compounds [47]. While CRISPR tools for Bifidobacteria have yet to be developed, Oh and van Pijkeren developed a method to perform genome editing in *Lactobacillus reuteri* ATCC PTA 6475 [48], a bacteria shown to have interesting immunomodulatory and antimicrobial properties [49,50]. Modifications can be introduced by recombination of a single stranded DNA oligonucleotide mediated by the RecT protein, followed by selection with Cas9. CRISPR-Cas9 mediated genome editing was also recently demonstrated in *Lactobacillus plantarum*, where putting the recombination template on a plasmid rather than providing it as single stranded DNA led to the best results [51]. Barrangou and colleagues have recently highlighted how CRISPR tools could be used to enhance therapeutic effects of lactic acid bacteria [52]. For instance, researchers are exploring strategies to enhance bile salt hydrolase activity to improve strain survival in the gut, or to modify surface layer-associated proteins to change their immunomodulatory properties.

Other bacteria of interest include the Clostridia, a diverse class of bacteria that include strains of industrial interest but also many commensals of the gut microbiome, of which a few—and most notoriously *Clostridium difficile*—can be opportunistic pathogens. Several reports have demonstrated the use of CRISPR tools to modify species of biotechnological interest including *Clostridium acetobutylicum*, *Clostridium beijerinckii* and *Clostridium cellulolyticum* [53–55]. CRISPR-Cas9 tools have now also enabled the engineering of *C. difficile* [56,57]. Note that non-toxigenic Clostridia, including non-toxigenic *C. difficile*, form part of the normal human gut microbiome [58] and could potentially be engineered as interesting probiotics. *Clostridium butyricum* MIYAIRI 588 has notably been developed as a probiotic against *C. difficile* infections [59], and could likely be engineered using the CRISPR tools developed for other Clostridium species.

Finally, CRISPR-Cas9 tools have also been developed for *Staphylococcus aureus* [60–62], an opportunistic pathogen commonly found on the skin [63]. Similarly to Clostridiae, Staphylococci can either be beneficial commensals or pathogens depending on the genetic makeup of specific strains. In addition to these microbiome-associated bacteria, CRISPR-Cas9 tools have been developed for other bacterial species including *Bacillus subtilis* [64]. In the future the catalogue of bacteria that can be engineered with CRISPR tools will likely continue to expand, enabling an increasing number of applications.

## 3. CRISPR editing of unicellular eukaryotes

While less studied than bacteria, commensal protozoans and fungi are highly prevalent in healthy populations [65,66], and some yeast can be used as probiotics in additive therapies. Most famously *Saccharomyces boulardii* was isolated by the French scientist Henri Boulard in 1923 with the purpose of controlling the symptoms of diarrhoea and is now commonly used as a probiotic. This yeast strain has been engineered with CRISPR-Cas9 to introduce various modifications, including the introduction of an exogenous metabolic pathway and the production of human lysozyme [67]. *Saccharomyces boulardii* has also been engineered with the help of CRISPR for the development of oral vaccines [68]. More generally, many CRISPR-Cas9 editing strategies have been developed in *Saccharomyces cerevisiae* [69,70] as well as microbiome-associated fungi, including the opportunistic pathogen *Candida albicans* [71,72]. Along the same line, CRISPR tools developed for protozoan parasites like *Plasmodium falciparum* [73,74] could likely be adapted to commensal protozoans like *Blastocystis*.

## 4. CRISPR editing of bacteriophages

Phages have been applied to cure bacterial infections, with many reported successes in various animal models of lung, skin or gut infection. Engineered phages present interesting subtractive therapeutic opportunities to treat infectious diseases and target the microbiome. In particular, phage adsorption elements like tail fibres and tail tips can be engineered to modify their host range [75]. Temperate phages can be engineered to remove potential virulence factors and turned into lytic phages to limit the risks associated with their use in phage therapy [76]. Phages can further be modified to disperse biofilm [77], encode antimicrobial proteins [78] or other functions of interest. Temperate phages that can be stably maintained in the bacterial cell as prophage or plasmid can be engineered during their lysogenic cycle using the tools described above. However, the modification of lytic phages is particularly challenging as they never reside as a stable genetic element in the cell and antibiotic selection markers cannot be used.

Strategies to edit lytic bacteriophages with CRISPR tools have now been developed. A guide RNA is designed to target the phage genome and a genetic modification of interest is typically cloned on a vector with homology arms to promote recombination with the phage DNA. Shortly after viral DNA entry, Cas nucleases cleave the target sequence and the lesion is repaired through recombination with the provided template, resulting in the edited phage. Only edited phages can then form plaques on bacteria carrying the CRISPR-Cas9 system. This strategy was first demonstrated using the type I-E CRISPR-Cas system of *E. coli* to engineer phage T7 [79], followed shortly thereafter by a demonstration that the CRISPR-Cas9 system from *Streptococcus thermophilus* could be used to edit virulent phages of this bacterium [80]. These techniques have now been extended to the engineering of *Lactococcus lactis* phage p2 [81], of phage T4 in *E. coli* [82] and phage vB_BsuP-Goe1 in *Bacillus subtilis* [83]. In one example, the type III CRISPR-Cas10 system from *Staphylococcus epidermidis* has also been used to edit staphylococcal phages [84]. Note that lytic phages can also be cloned and engineered in yeast [75], where CRISPR-Cas9 tools are readily available.

Beyond the possible use of natural or engineered bacteriophages to cure infections caused by specific pathogens, the recent description of their role as key components of the microbiome will likely open the way to new phage-based therapies [85]. Not only can phages alter the structure of the microbiome by infecting specific species, but they can also alter the genotype and phenotype of the bacteria they infect through horizontal gene transfer and lysogeny. As such, phages likely contribute to the maintenance of the intestinal homeostasis either in health or in disease (dysbiosis) [86]. The use of temperate phages to influence the composition and phenotype of bacteria in the microbiome could thus be viewed as an interesting modulatory therapeutic strategy, but a better understanding of these complex ecological interactions will be needed for the development of such therapies.

## 5. CRISPR antimicrobials

Besides their use to directly kill target bacteria, phages can be used as DNA delivery vectors. Plasmids carrying a phage packaging signal, known as phagemids, can be used to deliver various effector DNA circuits to target bacterial populations. Phagemids can be packaged into phage particles in the presence of a helper phage that carries all the elements necessary for the production of functional capsids that are missing from the phagemid DNA [87]. In addition, the helper phage can be modified in order to block packaging of its DNA [88]. The M13 phagemid was used to deliver various toxins or restriction enzymes to *E. coli* [89–91]. The Pf3 phage has also been used to a deliver a restriction enzyme, and successfully treat a *Pseudomonas* infection in mice [78].

CRISPR-Cas systems themselves can be delivered to populations of bacteria using this strategy with the purpose of specifically eliminating bacteria carrying target sequences in their genome. This strategy has already been demonstrated in *E. coli* [92] and in *S. aureus* [93]. In the first study, a plasmid carrying Cas9 and guide RNAs targeting antibiotic resistance genes were injected into bacterial populations using the M13 phagemid system. Efficient cell death was observed as expected when the target gene was present. In the second study, a phagemid based on *Staphylococcus* phage phiNM1 was constructed by cloning its packaging site on a plasmid carrying a CRISPR-Cas9 system. This phagemid was then tested against various antibiotic resistance genes and virulence factors. Both reports demonstrated the possibility of using CRISPR-Cas systems to eliminate a specific target bacterial genotype in a mixed population, both *in vitro* and *in vivo*, testing a wax worm infection model in the former case and a mouse skin colonization model in the second one. These studies also investigated the outcome of targeting a plasmid rather than the chromosome. Cas9 cleavage of a target plasmid leads to cell survival and plasmid loss. Note however that in cases where the plasmid carries a toxin–antitoxin addiction system, the cells will die as a consequence of plasmid loss.

In yet another study, it was proposed to use temperate phages rather than phagemids in order to introduce CRISPR-Cas systems in *E. coli* [94]. The CRISPR array was programmed to cure plasmids carrying antibiotic resistance genes, thereby sensitizing bacteria to antibiotics. In this work an additional trick was played: a lytic phage was engineered to carry sequences matching the guide RNAs encoded by the CRISPR array. This phage could then be used to kill bacteria that did not carry a functional CRISPR-Cas system and ensure the fixation of the CRISPR prophage in the population.

CRISPR-based antimicrobials offer the possibility to develop novel subtractive therapies, enabling the killing of bacteria based on their sequence without disturbing the rest of the microbiota. We now understand increasingly how different strains of a given bacterial species can have profoundly different effects on our health. The accessory genome that differentiates bacterial strains includes genomic islands, prophages and plasmids that impact the interaction of bacteria with each other and with their host. These elements frequently include virulence factors, toxins and antibiotic resistance genes [95,96]. CRISPR antimicrobials could become a powerful tool both to study the effect of specific strains by removing them from the microbiome, and to eliminate undesired strains as a therapeutic strategy. Note that many bacteria carry active CRISPR-Cas systems of their own that can also be harnessed to trigger self-targeting and cell death. The different approaches to CRISPR antimicrobials and the associated challenges have been reviewed in more detail elsewhere [97,98].

## 6. Controlling gene expression with CRISPR

Beyond the targeted elimination of strains and the genetic modification of commensals, CRISPR-Cas systems can also be engineered to modulate gene expression. These engineered CRISPR-Cas systems could be used to modulate the activity of bacteria used as probiotics in additive strategies, or could directly be delivered to the resident bacteria of the microbiome through transduction or conjugation (figure 2).

The Cas9 protein carries an HD nuclease domain and a RuvC nuclease domain, each cleaving the target DNA on a different strand [10]. Mutation of the catalytic residues abolishes DNA restriction while maintaining strong on-target binding. Binding of dCas9 to promoter sequences strongly inhibits the initiation of transcription, while binding inside transcribed regions can inhibit transcription elongation [99,100]. Note that dCas9 is only able to efficiently block the running RNA polymerase when it is guided by an RNA that binds to the non-template (coding) strand of DNA. The level of complementarity between the guide RNA and the target can be used to control the rate at which RNA polymerase ‘kicks out’ dCas9 from the target and completes transcription [33]. This mechanism can be used to precisely and robustly reduce gene expression by defined relative amounts, offering a powerful tool to modulate the physiology of target bacteria.

Gene silencing with dCas9, also known as CRISPRi, is comparatively much easier to perform than Cas9-mediated genome editing in bacteria. This has led to the rapid and broad adoption of this technology in a wide range of bacteria, including pathogenic streptococci [100,101], *Pseudomonas* [102], *Staphyloccoci* [103], *Mycobacteria* [104] and *Mycoplasma* [105], as well as a large number of bacterial species of industrial interest. Directly relevant to this review, dCas9 repression has also been performed in microbiome-associated bacteria like *Lactococcus lactis* [106] and *Bacteroides thetaiotaomicron*, where it was used to alter the bacterial metabolic capacity and its resistance to antimicrobial peptides [107]. In the latter work, the CRISPRi repression was also shown to be functional in the mouse gut.

It is also possible to repurpose dCas9 as a transcriptional activator, in a strategy also known as CRISPRa. In a first proof of concept, dCas9 was fused to the omega subunit of the RNA polymerase, yielding moderate activation [100]. In a recent study, activator domains were recruited to the dCas9 ribonucleic complex using a more elaborate strategy. Activator domains were fused to the MS2 coat protein, which binds to a MS2 hairpin itself fused to the guide RNA [108]. Several candidate activators could easily be tested in this manner and strong activation was obtained with SoxS, which could further be optimized to yield up to a 50-fold increase in the expression of a target reporter gene. The authors further showed how this design could be used to activate silent metabolic pathways. Note however that this strategy is very sensitive to the distance between the target and the promoter. Binding needs to occur within a narrow window roughly 60–90 bases upstream of the transcription start site. When no PAM is available in the desired range, it might not be possible to use this tool without further efforts to modify PAM specificity [109].

One appealing feature of CRISPRi is how it can easily be scaled to high-throughput genetic screens. Arrayed libraries of guide RNAs have already proven very useful to investigate the function of essential genes in *B. subtilis* and *S. pneumoniae* [101,110]. Potential caveats of using dCas9 were discovered in a recent study in which a pool of guide RNAs was used to target approximately 10^5^ positions in the chromosome of *E. coli* [111]. This study revealed that dCas9 can cause off-target effects at positions with as little as 9 nt of homology to the guide RNA. The same study also revealed an unexpected toxicity of dCas9 in *E. coli*. Among other important design rules, this work highlighted the importance of fine-tuning the concentration of dCas9 to avoid this toxicity while maintaining strong on-target repression. Using such an optimized expression cassette and relying on the information gathered by multiple guides targeting the same gene can enable powerful screens to be performed using dCas9 [112,113].

Other types of CRISPR-Cas systems can also be used to silence genes. In type I systems, the Cas3 nuclease is responsible for DNA degradation. In its absence, the multiprotein complex known as Cascade will bind target sequences without introducing any DNA damage. This can be used to block gene expression in the same manner as dCas9 [114,115]. The dead variants of the Cas12 (Cpf1) protein from type V system can also be used to block gene expression in bacteria, as already demonstrated with Cas12 proteins of various origins [116–118]. Finally gene silencing can also be achieved at the RNA level directly using the Cas13 protein from type VI systems, which acts as a RNA-guided RNAse [12,119]. Note however that Cas13 displays a non-specific RNAse activity once it has found its target. This triggers collateral RNA damage that is likely toxic to the cell and was seen to reduce cell growth in *E. coli* [12]. This collateral activity makes it impractical to silence genes in bacteria, but variants of Cas13 might be identified in the future that only carry a specific RNAse activity. Technologies have also been developed to edit RNA with a deactivated Cas13 fused to a base modification domain [120].

CRISPRi and CRISPRa have also been adapted to eukaryotic cells and—of particular relevance to this review—to *Saccharomyces cerevisiae* [121], where it could help in the development of probiotic yeast. Note that while dCas9 itself can efficiently block transcription in bacteria, CRISPRi in eukaryotic cells requires the fusion of dCas9 to effector domains.

## 7. Challenges and future prospects

Over the past few years, we have seen the application of CRISPR tools to organisms as diverse as Firmicutes, Proteobacteria, yeasts and human cells. The fact that the Cas9 protein from *S. pyogenes* worked easily in so many different cellular backgrounds was surprising to many. This bodes well for the future applications of CRISPR technologies to the extremely diverse set of organisms that compose our microbiome, even though Cas9 from different origins, or even other Cas proteins, might be better suited than others to specific organisms.

While CRISPR-Cas systems seem easily portable between species, one key consideration is that the outcome of DNA cleavage by Cas nucleases can be very different depending on the type and efficiency of the DNA repair pathways present in different organisms. As an example, Cas9 cleavage in the chromosome of mammalian or yeast cells leads to DNA repair through homologous recombination or non-homologous end joining, while the main outcome of efficient Cas9 cleavage in bacterial genomes is cell death. This is likely owing to the absence or poor efficiency of non-homologous end joining (NHEJ) systems in most bacterial species. Among bacteria that naturally carry NHEJ systems, Cas9 breaks could be repaired by NHEJ in some species but not others [55,122,123]. Efforts to import NHEJ systems into bacteria that lack them have yielded limited success [20,123,124]. Different bacterial species also carry more or less efficient homologous recombination systems. Bacteria that can undergo natural transformation and efficient homologous recombination can easily be modified using CRISPR-Cas9 tools, while other bacteria like *E. coli* require the use of an exogenous recombination system [26]. This heterogeneity in DNA repair capabilities between organisms means that specific tools and strategies need to be designed for different species. CRISPR tools that do not rely on DNA cleavage will likely be more easily portable. These include base editing and CRISPRi strategies.

One of the main obstacles for the broad adoption of these technologies to manipulate members of the microbiome is the difficulty of introducing exogenous DNA in many bacterial species. Some bacteria are more or less amenable to techniques such as electroporation, conjugation or transduction. Many bacteria also carry restriction systems that destroy incoming DNA. Others might not be able to replicate plasmid DNA if the origin of replication is not carefully chosen. Finally, bacteria that cannot be easily grown in the laboratory are obviously hard to engineer. These obstacles are not specific to CRISPR technologies and researchers have found ways to engineer many bacterial species through the construction of dedicated vectors. CRISPR tools thus need to be redesigned to fit the requirements of individual target species. Finding more standardized approaches and more universal tools to deliver DNA to bacteria would go a long way to accelerate research and engineering of a diverse set of bacteria. In a recent study, Peters and colleagues used conjugation and transposition for DNA delivery and integration in the bacterial chromosome to facilitate the use of CRISPRi in many species [125]. Beyond the use of CRISPR tools *in vitro*, conjugation as well as phage delivery systems enable the delivery of DNA directly *in vivo* and could be used for the development of novel therapies. Both transduction and conjugation also open the possibility of targetting non-culturable bacteria directly in their natural environment. Phage particles can be used to deliver DNA efficiently into specific bacterial strains, however this specificity is such that dedicated phage capsids will likely have to be engineered for each therapeutic indication. The host range of bacteriophage capsids can be extended through the modification of the proteins that interact with the bacterial envelope such as the tail fibres [75]. In a recent study, Qimron and colleagues employed a directed evolution approach to extend the host range of bacteriophage particles for DNA transduction [126]. This type of approach will be critical to engineer delivery vectors for a wide range of target bacteria, but whether transduction or conjugation can efficiently reach the right bacteria in the complex spatial structure of the microbiome largely remains to be investigated.

The increasing knowledge of the profound impact that the microbiome has on human health is driving the development of novel therapeutic avenues to treat infectious disease, metabolic disease, immune disease and even neurological disorders. However, the outcome of current therapies that target the microbiome is often uncertain owing to our limited understanding of the complex ecological interactions that occur within microbial communities and with the host immune system. CRISPR tools provide exciting strategies not only to study the biology of microbes, but also to elucidate their role within complex communities and drive the development of novel therapies.

## Data accessibility

This article has no additional data.
